# Hyperacusis in Tinnitus Individuals Is Associated with Smaller Gray Matter Volumes in the Supplementary Motor Area Regardless of Hearing Levels

**DOI:** 10.3390/brainsci14070726

**Published:** 2024-07-19

**Authors:** Punitkumar Makani, Marc Thioux, Elouise A. Koops, Sonja J. Pyott, Pim van Dijk

**Affiliations:** 1 Department of Otorhinolaryngology—Head and Neck Surgery, University of Groningen, University Medical Center Groningen, 9700 RB Groningen, The Netherlands; p.makani@umcg.nl (P.M.); ekoops@mgh.harvard.edu (E.A.K.); s.pyott@umcg.nl (S.J.P.); p.van.dijk@umcg.nl (P.v.D.); 2 Graduate School of Medical Sciences (Research School of Behavioral and Cognitive Neurosciences), University of Groningen, 9713 AV Groningen, The Netherlands; 3 Department of Radiology, Massachusetts General Hospital, Harvard Medical School, Boston, MA 02114, USA

**Keywords:** gray matter, hearing loss, hyperacusis, supplementary motor area, tinnitus, voxel-based morphometry

## Abstract

Recent evidence suggests a connection between hyperacusis and the motor system of the brain. For instance, our recent study reported that hyperacusis in participants with tinnitus and hearing loss is associated with smaller gray matter volumes in the supplementary motor area (SMA). Given that hearing loss can affect gray matter changes in tinnitus, this study aimed to determine if the changes reported in our previous findings of smaller SMA gray matter volumes in hyperacusis persist in the absence of hearing loss. Data for this study were gathered from four prior studies conducted between 2004 and 2019 at the University Medical Centre Groningen (UMCG). A total of 101 participants with tinnitus and either clinically normal hearing (normal hearing with tinnitus or NHT, n = 35) or bilateral sensorineural hearing loss (hearing loss with tinnitus or HLT, n = 66) were included across four studies. Hyperacusis was determined by a score of ≥22 on the Hyperacusis Questionnaire (HQ). In the NHT group, 22 (63%) participants scored ≥22 on the HQ (NHT with hyperacusis: mean age 44.1 years, 12 females), while in the HLT group, 25 (38%) participants scored ≥22 on the HQ (HLT with hyperacusis: mean age 59.5 years, 10 females). The 2 × 2 between-group ANOVAs revealed that hyperacusis is associated with smaller SMA gray matter volumes, regardless of hearing levels. Notably, the smaller SMA gray matter volumes in hyperacusis were primarily influenced by the attentional subscales of the HQ. The association between hyperacusis and the motor system may indicate a constant alertness to sounds and a readiness for motor action.

## 1. Introduction

Hyperacusis is a condition characterized by an increased sensitivity to everyday environmental sounds of mild to moderate intensity. Hyperacusis can significantly impact the quality of life of those affected [1,2,3]. While the exact prevalence of hyperacusis remains unknown, it is estimated to affect up to 17% of the general adult [4] and pediatric [5] population. It occurs significantly more often in individuals with hearing disorders, such as tinnitus and hearing loss. In fact, approximately 90% of individuals with hyperacusis report suffering from tinnitus [1], and, vice versa, approximately 63% of individuals with tinnitus report concurrent hyperacusis [4]. Additionally, an estimated 59% of individuals with hyperacusis also suffer from hearing loss [6]. In addition to tinnitus and hearing loss, there are other factors that can contribute to hyperacusis in both adults and children, such as head injuries, Williams syndrome, upper respiratory tract infections, chronic otitis media, and obsessive-compulsive disorder [2,7,8].

At a mechanistic level, increased central gain, a homeostatic response whereby the auditory neurons exhibit an increased response to a given input, could explain hyperacusis [9]. Functional neuroimaging studies have provided support for this theory, demonstrating that individuals with hyperacusis show increased sound-evoked activity in the auditory pathway [10,11,12]. Moreover, hyperacusis may also involve non-auditory brain regions, such as the orbitofrontal cortex, the anterior cingulate cortex, and the supplementary motor area [13,14]. In particular, in a recent whole-brain voxel-based morphometry (VBM) study, we observed that in participants with tinnitus and hearing loss, concurrent hyperacusis was associated with a smaller gray matter volume in the right supplementary motor area (SMA) [14]. Since the SMA is a brain region involved in action initiation, this finding suggests that these changes in the gray matter there might be related to a motor hyper-reactivity to sounds. In support of this hypothesis, a resting state (electroencephalogram) EEG by Song et al. demonstrated increased beta waves with a generator located in the SMA in individuals with hyperacusis [13].

In our previous VBM study, all participants had tinnitus and hearing loss [14]. This study design allowed us to identify gray matter differences that are specifically associated with hyperacusis, as the two groups were matched for tinnitus and hearing loss. Hyperacusis is, however, also diagnosed in individuals with clinically normal hearing thresholds. In this population, it is thought that hidden hearing loss, with various possible underlying etiologies, could induce hyperacusis and tinnitus [15]. In a recent meta-analysis, we established that the presence or absence of hearing loss can significantly influence gray matter volumes in participants with tinnitus [16]. By including a group with tinnitus and clinically normal hearing thresholds, the current study aimed to clarify whether smaller SMA gray matter volumes are attributable to hyperacusis alone or if they are influenced by the presence or absence of hearing loss.

In this retrospective case-control study, therefore, we examined whether SMA gray matter volumes were smaller in hyperacusis adults with tinnitus and clinically normal hearing. If the SMA gray matter volume differences associated with hyperacusis are independent of hearing status, then individuals with clinically normal hearing and hyperacusis should show the same trend as their counterparts with hearing loss. To test this hypothesis, we conducted 2 × 2 between-group ANOVAs (hyperacusis × hearing loss) within independently defined volumes of interest in the SMA. With this approach, we aim to examine the potential interactions between the effects of hyperacusis and hearing loss on gray matter volumes in the SMA region. Ultimately, we hope that these findings will offer insights into the structural mechanisms of hyperacusis, aiding the identification of structural biomarkers for more targeted and effective interventions for individuals suffering from hyperacusis, ultimately improving their quality of life.

## 2. Materials and Methods

Data for this study were gathered from four prior neuroimaging studies conducted between 2004 and 2019 at the Department of Otorhinolaryngology of the University Medical Centre Groningen (UMCG) [17,18,19,20]. These studies were conducted in accordance with the Declaration of Helsinki and were approved by the UMCG’s medical ethics committee (METc), which additionally granted approval for data reanalysis (METc number: 2020/347). All participants provided written informed consent.

### 2.1. Participants

A total of 101 participants with tinnitus and either clinically normal hearing (the normal hearing with tinnitus or NHT group, n = 35) or bilateral sensorineural hearing loss (the hearing loss with tinnitus or HLT group, n = 66) were included. Pure tone air-conduction audiometry was utilized to assess hearing thresholds across octave frequencies ranging from 0.25 to 8 kHz. Hearing loss was defined as an average hearing threshold (or pure tone average—PTA) of ≥25 dB HL across all tested frequencies (0.25, 0.5, 1, 2, 4, and 8 kHz) for both ears. Hyperacusis was evaluated using the 14-item Hyperacusis Questionnaire (HQ) [21]. Additionally, the tinnitus burden was quantified using the 25-item Tinnitus Handicap Inventory (THI) [22]. Anxiety and depression levels were determined using the 14-item Hospital Anxiety and Depression Scale (HADS) [23]. Handedness was assessed with the 10-item Edinburgh Handedness Inventory (EHI) [24].

Similar to our previous study [14], a cut-off score of ≥22 on the Hyperacusis Questionnaire was used to distinguish the presence or absence of hyperacusis [25].

### 2.2. MRI Data Acquisition

For each participant, high-resolution 3-dimensional T1-weighted anatomical brain images were obtained using a 3-Tesla Philips Intera scanner (Philips Medical System, Best, The Netherlands) equipped with either an 8-channel [17,18,19] or 32-channel [20] phase-array head coil (SENSE). Fast-field echo images were acquired, with a reconstructed voxel size of 1 × 1 × 1 mm, using the acquisition parameters outlined in Table S1 in the Supplementary Materials.

### 2.3. MRI Data Preprocessing

T1-weighted anatomical brain images were preprocessed using the CAT12.7 toolbox (v.1653, Structural Brain Mapping Group, University of Jena, Germany, http://dbm.neuro.uni-jena.de/cat12/, accessed on 11 July 2024) within SPM12 (v.7487, Wellcome Centre for Human Neuroimaging, University College London, https://www.fil.ion.ucl.ac.uk/spm/, accessed on 11 July 2024), running on MATLAB R2020a (The MathWorks, Natick, MA, USA). Firstly, the brain images underwent correction for bias, noise, and global intensity variations. Subsequently, the brain images were normalized to the Montreal Neurological Institute (MNI152) template, with voxel dimensions resampled to 1.5 × 1.5 × 1.5 mm. During normalization, the brain image was segmented into gray matter, white matter, and cerebrospinal fluid. The segmented gray matter images were then modulated using the Jacobian determinant [26,27]. Finally, recommended quality control measures were used to assess the quality of the resulting normalized and modulated gray matter images. More details can be found in our previous study [14].

### 2.4. Volume-of-Interest Selection for the Supplementary Motor Area

Similar to our previous study [14], we selected the volume of interest (VOI) corresponding to the supplementary motor area (SMA), as defined in a previous study, using both anatomical as well as functional MRI data [28]. This meta-analysis utilized both task-related activation coordinates and anatomical boundaries to delineate the motor cortex areas. This SMA VOI is freely accessible as the Human Motor Area Template (HMAT) atlas from http://lrnlab.org/ (accessed on 11 July 2024). More details can be found in Figure S1 in the Supplementary Materials.

In order to deepen our investigation into SMA gray matter in hyperacusis, we also investigated VOIs corresponding to the anterior and posterior subdivisions of the SMA (SMA_A6m and SMA_A4ll respectively), as defined by the Human Brainnetome atlas based on the structural and functional connectome architecture [29]. These SMA VOIs are freely accessible as the Human Brainnetome (HBN) atlas from https://atlas.brainnetome.org/bnatlas.html (accessed on 11 July 2024). More details can be found in Figure S1.

### 2.5. Gray Matter Extraction from Volume-of-Interest Areas

First, the selected VOIs were resampled to 1.5 × 1.5 × 1.5 mm voxel dimensions to align with the voxel dimensions of normalized and modulated gray matter images. Next, gray matter volumes were extracted from each selected VOI for every participant using the “get_totals” MatLab script. This MatLab script is freely available for download from http://www0.cs.ucl.ac.uk/staff/g.ridgway/vbm/get_totals.m/ (accessed on 18 July 2024). Subsequently, these extracted gray matter volumes were subjected to further analyses using SPSS (v.29, IBM Corp., Armonk, New York, NY, USA).

### 2.6. Statistical Analyses

#### 2.6.1. Demographic, Audiometric, and Questionnaire Data

The statistical analyses of demographic, audiometric, and questionnaire data were performed using SPSS29. To assess the between-group differences for continuous variables, a nonparametric independent sample one-way ANOVA (Kruskal–Wallis H test) was used, followed by Bonferroni-corrected post hoc *t*-test analysis. For categorical variables, the chi-square test was used to examine between-group differences. A statistical threshold of *p* ≤ 0.05 was used for all analyses.

#### 2.6.2. Gray Matter Volume of the Supplementary Motor Area in Association with Hyperacusis

##### Effects of Hyperacusis and Hearing Loss on SMA Gray Matter Volume

We performed 2 × 2 between-group ANOVAs to investigate the main effects of hyperacusis and hearing loss, as well as the effect of their interaction on the gray matter volumes of the selected SMA VOIs. Age, handedness scores, and TIV were included as covariates. The statistical threshold for these analyses was set at *p* ≤ 0.05. We tested for group differences in the HMAT left and right SMA VOI. To extend our analysis, we repeated the analysis in the posterior and anterior portions of the left and right SMA (see Section 2.4 and Figure S1).

##### Differentiating the Effects of the Attentional and Social Subscales of the Hyperacusis Questionnaire on SMA Gray Matter Volumes

Responses to the Hyperacusis Questionnaire can be classified into two component groups: attentional and social [30]. To assess whether smaller SMA gray matter volumes were (more) influenced by the scores for either the attentional or social component of the HQ, Spearman’s rank correlation coefficients between the SMA gray matter volumes (HMAT and HBN VOIs) and scores on the attentional and social subscales of the HQ were computed across all participants. The statistical threshold for these analyses was set at *p* ≤ 0.05.

##### Effects of Anxiety, Depression, or Tinnitus Burden

To assess the impact of anxiety, depression, and tinnitus burden on SMA gray matter volumes (HMAT and HBN VOIs), Spearman’s rank correlation coefficients between the gray matter volumes of the selected SMA VOIs and either the HADS (anxiety and depression) or the THI (tinnitus burden) scores were computed across all participants. The statistical threshold for these analyses was set at *p* ≤ 0.05.

## 3. Results

### 3.1. Demographic, Audiometric, and Questionnaire Data

**Figure 1:**
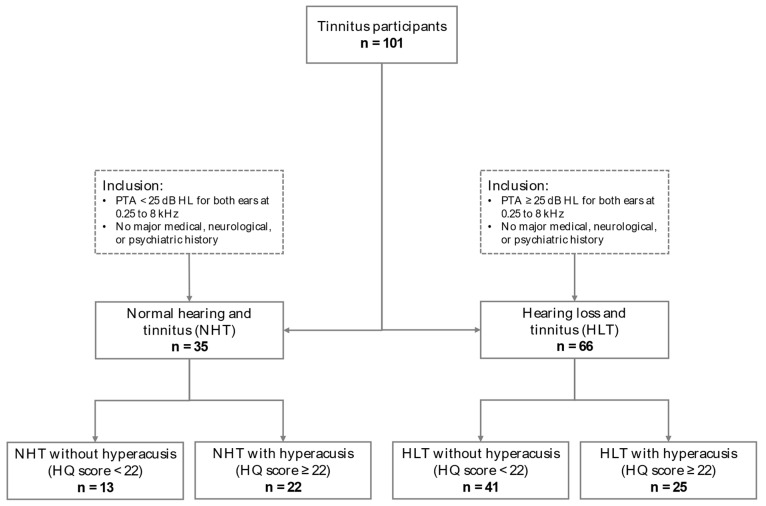
Flowchart of participant inclusion criteria for the four participant groups. For all participants, pure tone air-conduction audiometry was utilized to assess hearing thresholds across octave frequencies ranging from 0.25 to 8 kHz. Hearing loss was defined as an average hearing threshold (or pure tone average—PTA) of ≥25 dB HL across all tested frequencies (0.25, 0.5, 1, 2, 4, and 8 kHz) for both ears. A cut-off score of ≥22 on the Hyperacusis Questionnaire (HQ) was used to distinguish the presence or absence of hyperacusis.

Participants were classified in the hyperacusis groups if they scored above 22 on the Hyperacusis Questionnaire [14,21,25]. Accordingly, in the NHT group, 22 out of 35 participants (63%) were classified as having hyperacusis (NHT with hyperacusis: mean age ± standard deviation = 44.1 ± 12.2 years, 12 females). The remaining 13 participants who scored below the cut-off score were assigned to the control group (NHT without hyperacusis: mean age ± standard deviation = 45.5 ± 11.2 years, 5 females). In the HLT group, 25 out of 66 participants (38%) scored above the cut-off score for hyperacusis (HLT with hyperacusis: mean age ± standard deviation = 59.5 ± 7.9 years, 10 females). The remaining 41 participants who scored below the cut-off score were assigned to the control group (HLT with hyperacusis: mean age ± standard deviation = 58.3 ± 10.5 years, 9 females). Participant inclusion criteria for the four groups are shown in Figure 1. The proportion of individuals suffering from hyperacusis was significantly higher in the group with clinically normal hearing thresholds, compared to the group with hearing loss [*X*^2^(1) = 5.7, *p* = 0.017].

**Figure 2:**
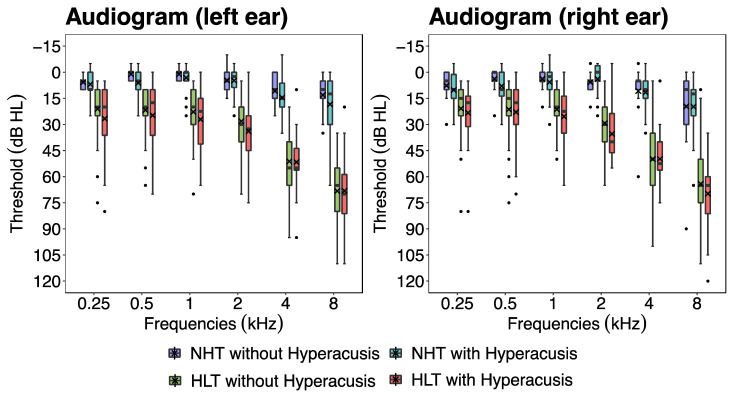
Box plots showing the hearing thresholds for the four participant groups. Participants with hearing loss (the HLT group either with or without hyperacusis) had worse hearing thresholds than those with clinically normal hearing (the NHT group either with or without hyperacusis) [*X*^2^(3) = 30.0, *p* < 0.001]. The crosses added to the box plots represent the mean values. HLT, hearing loss and tinnitus; NHT, normal hearing and tinnitus.

These four groups showed significant differences across several variables. The statistical analyses revealed significant between-group differences in age [*X*^2^(3) = 30.0, *p* < 0.001], hearing thresholds [PTA 0.25 to 8 kHz: *X*^2^(3) = 65.8, *p* < 0.001], HADS scores [HADS-anxiety: *X*^2^(3) = 11.0, *p* < 0.001; HADS-depression: *X*^2^(3) = 8.4, *p* < 0.001]; and THI scores [*X*^2^(3) = 13.0, *p* < 0.001]. Participants with hearing loss (the HLT group either with or without hyperacusis) were, on average, older than those with clinically normal hearing (the NHT group either with or without hyperacusis). Mean hearing thresholds for both ears at octave frequencies ranging from 0.25 to 8 kHz per group are shown in Figure 2. Summarized data for the four groups are shown in Table 1 (more details are provided in Table S2 in the Supplementary Materials).

### 3.2. Association between Hyperacusis and SMA Gray Matter Volumes

#### 3.2.1. Human Motor Area Template (HMAT) Atlas Analysis

**Figure 3:**
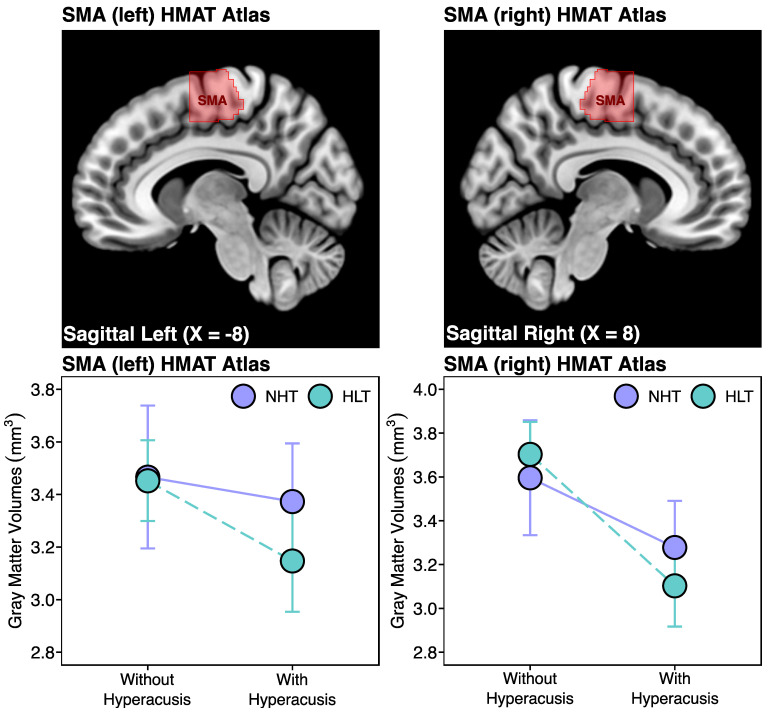
The SMA VOIs were derived from the Human Motor Area Template (HMAT) atlas (upper panels). The 2 × 2 between-group ANOVAs (corrected for age, handedness scores, and total intracranial volume) revealed that hyperacusis has a significant negative main effect on the right supplementary motor area (SMA) gray matter volumes [SMA right: F(1,94) = 21.0, *p* < 0.001]. There was no significant main effect of hearing loss, nor any interaction between the effects of hyperacusis and hearing loss, on the bilateral SMA gray matter volumes. The presence of hyperacusis was defined by a cut-off score of ≥22 on the 14-item HQ. The graphs show the mean and 95% confidence interval (CI). HLT, hearing loss and tinnitus; NHT, normal hearing and tinnitus.

Within the right HMAT SMA VOIs, the 2 × 2 between-group ANOVAs revealed a significant main effect of hyperacusis on gray matter volumes [F(1,94) = 21.0, *p* < 0.001], with participants scoring above the threshold for hyperacusis and displaying lower SMA gray matter volumes relative to the controls (Figure 3). There was also a trend toward a negative main effect of hyperacusis in the left hemisphere [F(1,94) = 3.7, *p* = 0.058] (Figure 3). However, we found no significant main effect of hearing loss [SMA left: F(1,94) = 1.0, *p* = 0.329; SMA right: F(1,94) = 0.8, *p* = 0.774], nor any interaction between the effects of hyperacusis and hearing loss [SMA left: F(1,94) = 1.1, *p* = 0.298; SMA right: F(1,94) = 2.1, *p* = 0.152].

#### 3.2.2. Human Brainnetome (HBN) Atlas Analysis

**Figure 4:**
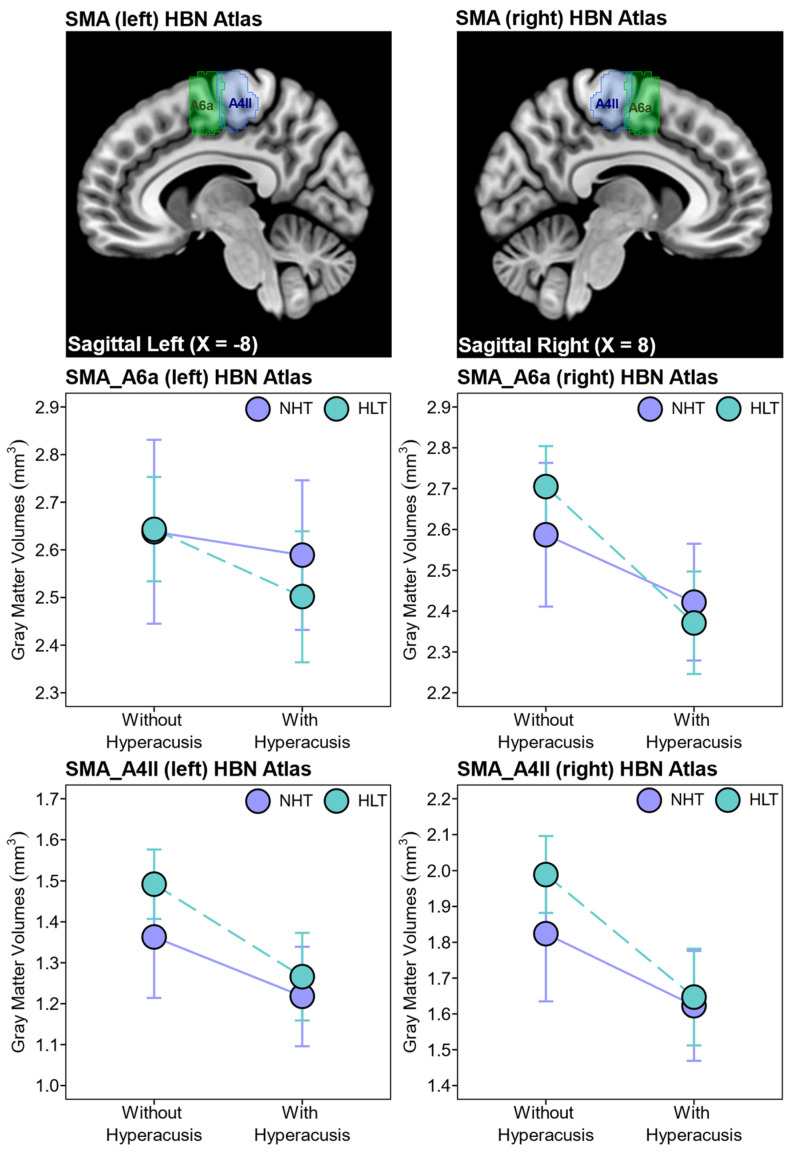
The SMA VOIs were derived from the Human Brainnetome (HBN) atlas (upper panels). The 2 × 2 between-group ANOVAs (corrected for age, handedness scores, and total intracranial volume) revealed that hyperacusis has a significant negative main effect on the right anterior (A6a) SMA gray matter volumes [SMA_A6a right: F(1,94) = 21.0, *p* < 0.001] and the bilateral posterior (A4ll) SMA) gray matter volumes [SMA_A4ll left: F(1,94) = 10.5, *p* < 0.001; SMA_A4ll right: F(1,94) = 14.1, *p* < 0.001]. There was no significant main effect of hearing loss nor any interaction between the effects of hyperacusis and hearing loss on the bilateral anterior/posterior SMA gray matter volumes. The presence of hyperacusis was defined by a cut-off score of ≥22 on the 14-item HQ. The graphs show the mean and 95% confidence interval (CI). HLT, hearing loss and tinnitus; NHT, normal hearing and tinnitus.

To further examine the effect of hyperacusis on SMA gray matter volumes, we repeated the analysis using the Human Brainnetome (HBN) atlas, which distinguishes between the anterior and the posterior SMA. For the anterior SMA (SMA_A6m) VOIs, the 2 × 2 between-group ANOVAs revealed a significant negative main effect of hyperacusis on the right anterior SMA gray matter volumes [F(1,94) = 13.7, *p* < 0.001], but no significant main effect on the left anterior SMA VOI [F(1,94) = 1.7, *p* = 0.200] (Figure 4). There was no significant main effect of hearing loss [SMA_A6m left: F(1,94) = 0.2, *p* = 0.637; SMA_A6m right: F(1,94) = 0.2, *p* = 0.675], nor any interaction between the effects of hyperacusis and hearing loss [SMA_A6m left: F(1,94) = 0.4, *p* = 0.521; SMA_A6m right: F(1,94) = 1.6, *p* = 0.203] on the anterior (A6m) SMA gray matter volumes.

For the posterior SMA (SMA_A4ll) VOIs, the 2 × 2 between-group ANOVAs revealed a significant negative main effect of hyperacusis on the posterior SMA gray matter volumes in both hemispheres [SMA_A4ll left: F(1,94) = 10.5, *p* < 0.001; SMA_A4ll right: F(1,94) = 14.1, *p* < 0.001] (Figure 4). Again, no significant main effect of hearing loss [SMA_A4ll left: F(1,94) = 1.7, *p* = 0.193; SMA_A4ll right: F(1,94) = 1.2, *p* = 0.269] and no significant interaction between the effects of hyperacusis and hearing loss [SMA_A4ll left: F(1,94) = 0.5, *p* = 0.478; SMA_A4ll right: F(1,94) = 1.1, *p* = 0.323] were observed on the posterior (A4ll) SMA gray matter volumes.

#### 3.2.3. Smaller Gray Matter Volumes in Hyperacusis Are Associated with the Attentional Subscale of the Hyperacusis Questionnaire

As expected, participants with hyperacusis scored higher on both the attentional and social subscales of the HQ, compared to participants without hyperacusis (Table 1). Interestingly, significant associations were only observed between the SMA gray matter volumes (HMAT and HBN VOIs) and the attentional subscale of the HQ (Table 2).

#### 3.2.4. Anxiety, Depression, or Tinnitus Burden Scores Do Not Drive Smaller SMA Gray Matter Volumes in Hyperacusis

Significant between-group differences were found in the HADS anxiety and depression and the THI tinnitus burden scores (Table 1). However, we found no significant relationship between the SMA gray matter volumes (HMAT and HBN VOIs) and either the HADS anxiety and depression scores or the THI tinnitus burden score (all *p* ≥ 0.284; more details are provided in Table S3 in the Supplementary Materials).

## 4. Discussion

In this study, we investigated whether the previously observed association between hyperacusis and smaller gray matter volumes in the right supplementary motor area (SMA) in individuals with bilateral sensorineural hearing loss and tinnitus would also be found in individuals with tinnitus and clinically normal hearing. Our findings confirmed that smaller SMA gray matter volumes related to hyperacusis are also present in individuals with tinnitus and clinically normal hearing. We did not find any interaction between hearing loss and hyperacusis on the SMA gray matter volumes while controlling for age. In other words, the smaller SMA gray matter volumes were associated with hyperacusis and not hearing loss.

The SMA functions in the fields of action preparation, initiation, and inhibition and is an integral part of the motor network [31,32]. Neurons in this area fire prior to the onset of an action, and a subset of those neurons responds to specific input modalities (visual, tactile, or auditory) when matched to a motor response [33,34]. Our previous work showed that the SMA is the only brain region to exhibit structural gray matter differences between groups associated with hyperacusis in tinnitus, and SMA gray matter volumes could classify participants with or without hyperacusis with high accuracy [14]. The current work extends our previous findings and reveals that the reduction in the SMA gray matter volumes in individuals with hyperacusis is not the result of hearing loss and may, therefore, reflect specific changes in the motor response to the perceived loudness of sound. In this study, we also found that smaller SMA gray matter volumes were primarily associated with the attentional subcategory of hyperacusis rather than the social subscale, suggesting that hyperacusis is linked to increased alertness regarding sound. There may be a correspondence between this heightened alertness and a constant readiness to provide a motor response to sound.

Our findings are consistent with previous reports in both animal models and humans. A recent study in mice has connected increased gain along the auditory pathway to involuntary facial movements [35], which may be related to the structural changes reported here. Supporting evidence from animal studies shows that hyperacusis-induced animals respond faster to auditory stimuli, indicating a link between motor activity and hyperacusis [9,36]. Additionally, a study in humans found that facial reaction and pupil dilatation to emotional sounds could distinguish between participants with or without hyperacusis and tinnitus who have clinically normal hearing thresholds [37]. Furthermore, an EEG study found increased functional activity in the SMA region in hyperacusis participants [13]. Similarly, participants with misophonia, a condition where very specific sounds (e.g., chewing or breathing) provoke strong emotional and physiological reactions, showed increased SMA activity when listening to triggering sounds relative to any other sound, as measured with an fMRI [38,39]. In addition, participants with obsessive-compulsive disorder (OCD) may exhibit heightened sensitivity to sound, which can lead them to develop fixations on avoiding certain sounds [40,41]. Interestingly, OCD has been closely linked with functional changes, including increased activation, in the SMA region [42,43]. Together, these findings and our study suggest a close association between hyperacusis and the brain network that is involved in preparing and conducting motor movements.

This study has several limitations. First, this study included a limited number of participants with tinnitus and clinically normal hearing and a limited number of female participants. These factors may affect the comparison and generalizability of the findings. Future research should aim to include larger and more sex-balanced groups. Second, the study utilized subjective hyperacusis questionnaires. Although these questionnaires are well-validated [25], future research should consider incorporating objective methods, such as psychoacoustic measurements [44], to precisely describe and quantify hyperacusis. Third, air-conduction tone audiometry was used to define hearing loss in this study. Future studies could also measure bone-conduction thresholds in order to explicitly differentiate between conductive and sensorineural hearing loss. Lastly, future research should also investigate the effects of the duration of hyperacusis, tinnitus, and hearing loss on changes in gray matter volumes. Only a few studies have previously examined the impact of tinnitus duration on gray matter volumes [45,46]. To our knowledge, no study has yet explored changes in response to the duration of hyperacusis or hearing loss (although the onset of the problem is often difficult to identify). These limitations highlight areas where future research could improve the study design to enhance the validity of findings related to gray matter changes in hyperacusis.

## 5. Conclusions

Our study highlights the importance of understanding the structural mechanisms underlying hyperacusis, and particularly the role of the SMA in the attentional control of auditory-motor coordination. By identifying specific brain regions associated with hyperacusis, we can develop more targeted and effective interventions to improve the quality of life for those affected by this condition. Future research should continue to explore the complex interactions between hyperacusis, tinnitus, and hearing loss, as well as the potential for neuroplasticity-based therapies to mitigate the impact of hyperacusis on daily functioning.

## Figures and Tables

**Table 1:** Overview of demographic, audiometric, and questionnaire data for the four participant groups.

Group	Normal Hearing and Tinnitus (NHT)		Hearing Loss and Tinnitus (HLT)		Statistic
	Without Hyperacusis	With Hyperacusis	Without Hyperacusis	With Hyperacusis	
**Demographic**					
n	13	22	41	25	-
Mean Age (years)	45.5 ± 11.2 (29–62) **^c,d^**	44.1 ± 12.2 (19–59) **^c,d^**	58.3 ± 10.5 (27–76) **^a,b^**	59.5 ± 7.9 (41–73) **^a,b^**	***X*^2^(3) = 30.0, *p* < 0.001**
Sex (male|female)	8|5	10|12	32|9	15|10	*X*^2^(3) = 7.0, *p* = 0.071
**Audiometric (for both ears 0.25 to 8 kHz)**					
Mean PTA (dB HL)	7.3 ± 5.4 **^c,d^**	9.4 ± 7.3 **^c,d^**	35.0 ± 8.6 **^a,b^**	38.2 ± 12.3 **^a,b^**	***X*^2^(3) = 65.8, *p* < 0.001**
**Questionnaires**					
HQ	16.5 ± 3.6 (10–21) **^b,d^**	27.1 ± 4.1 (22–35) **^a,c^**	13.2 ± 5.4 (0–21) **^b,d^**	26.2 ± 3.7 (22–33) **^a,c^**	***X*^2^(3) = 76.0, *p* < 0.001**
HQ-Attentional ^‡^	9.2 ± 2.3 **^b,d^**	13.5 ± 2.6 **^a,c^**	6.5 ± 2.7 **^b,d^**	13.5 ± 2.6 **^a,c^**	***X*^2^(3) = 57.5, *p* < 0.001**
HQ-Social ^‡^	1.8 ± 1.0 **^b,d^**	5.9 ± 2.7 **^a,c^**	2.6 ± 2.4 **^b,d^**	5.2 ± 2.0 **^a,c^**	***X*^2^(3) = 31.3, *p* < 0.001**
HADS-Anxiety	6.8 ± 4.2 (0–16)	6.5 ± 3.4 (2–15) **^c^**	3.7 ± 3.0 (0–11) **^b^**	5.9 ± 4.6 (0–16)	***X*^2^(3) = 11.0, *p* = 0.011**
HADS-Depression	5.0 ± 3.8 (0–14)	4.9 ± 3.7 (0–15)	3.2 ± 3.1 (0–10) **^d^**	6.2 ± 4.7 (0–16) **^c^**	***X*^2^(3) = 8.4, *p* = 0.038**
THI	33.7 ± 20.4 (4–66)	44.4 ± 20.5 (10–88) **^c^**	27.4 ± 19.3 (4–80) **^b,d^**	41.8 ± 20.8 (6–82) **^c^**	***X*^2^(3) = 13.0, *p* = 0.005**

Mean ± standard deviation (range). dB HL, decibel hearing loss; HADS, hospital anxiety depression scale; HQ, hyperacusis questionnaires; PTA, pure tone average (0.25 to 8 kHz); THI, tinnitus handicap inventory. **^a^** The group differed significantly (*p* ≤ 0.05) from the NHT group without hyperacusis. **^b^** The group differed significantly (*p* ≤ 0.05) from the NHT group with hyperacusis. **^c^** The group differed significantly (*p* ≤ 0.05) from the HLT group without hyperacusis. **^d^** The group differed significantly (*p* ≤ 0.05) from the HLT group with hyperacusis. ^‡^ The attentional and social subscales corresponding to the HQ were defined according to a previous study [30].

**Table 2:** Results of Spearman’s rank correlation coefficients between the SMA gray matter volumes (HMAT and HBN VOIs) and either the attentional or social subscales scores of the HQ across all participants. The SMA VOIs are shown in Figure S1 in the Supplementary Materials. The statistical threshold for Spearman’s rank correlation coefficients was set at *p* ≤ 0.05.

	HQ-Attentional Score	HQ-Social Score
**HMAT VOIs**		
SMA left	**r_s_(101) = −0.26, *p* = 0.010**	r_s_(101) = 0.10, *p* = 0.327
SMA right	**r_s_(101) = −0.33, *p* < 0.001**	r_s_(101) = −0.11, *p* = 0.273
**HBN VOIs**		
SMA_A6m left	r_s_(101) = −0.17, *p* = 0.089	r_s_(101) = 0.13, *p* = 0.210
SMA_A6m right	**r_s_(101) = −0.30, *p* = 0.001**	r_s_(101) = −0.06, *p* = 0.569

SMA_A4ll left	**r_s_(101) = −0.32, *p* = 0.001**	r_s_(101) = −0.11, *p* = 0.259
SMA_A4ll right	**r_s_(101) = −0.35, *p* < 0.001**	r_s_(101) = −0.14, *p* = 0.165

HBN, Human Brainnetome atlas; HMAT, Human Motor Area Template atlas; HQ, Hyperacusis Questionnaires; SMA, supplementary motor area; SMA_A6ma, anterior subdivision supplementary motor area; SMA_A4ll, posterior subdivision supplementary motor area; VOI, volume of interest.

## Data Availability

The datasets analyzed in the study are available from the corresponding author upon reasonable request. The datasets are not publicly available, due to privacy and ethical restrictions.

## Supplementary Materials

The following supporting information can be downloaded at: https://www.mdpi.com/article/10.3390/brainsci14070726/s1, Table S1: Acquisition parameters for T1-weighted anatomical brain imaging; Table S2: Overview of demographic, audiometric, questionnaire, and morphometric data for the four participant groups; Table S3: Results of Spearman’s rank correlation coefficients between the SMA gray matter volumes (HMAT and HBN atlas VOIs) and either the HADS anxiety and depression scores or the THI tinnitus burden scores across all participants. The statistical threshold for Spearman’s rank correlation coefficients was set at *p* ≤ 0.05. The SMA VOIs (HMAT and HBN atlas VOIs) are shown in Supplementary Figure S1; Figure S1: We selected the volume-of-interest (VOI) areas for the supplementary motor area (SMA), as defined in a previous study, using both anatomical and functional MRI data. These SMA VOIs (highlighted in red) are freely accessible as the Human Motor Area Template (HMAT) atlas from http://lrnlab.org/ (accessed on 11 July 2024). We also selected VOIs for the anterior and posterior subdivisions of the SMA, labeled as SMA_A6m (highlighted in green) and SMA_A4ll (highlighted in blue), respectively, as defined by the structural and functional connectome architecture. These SMA VOIs are freely accessible as the Human Brainnetome (HBN) atlas from https://atlas.brainnetome.org/bnatlas.html (accessed on 11 July 2024).
