# Supplementary material for: Hyperacusis in Tinnitus Individuals Is Associated with Smaller Gray Matter Volumes in the Supplementary Motor Area Regardless of Hearing Levels
Source: Brain Sci. 2024 Jul 19;14(7):726. doi: 10.3390/brainsci14070726 (PMC11275185; doi:10.3390/brainsci14070726)
Supplement: Supplementary file 1 [file brainsci-14-00726-s001.zip › brainsci-3087174-supplementary.pdf]

Supplementary Material

Hyperacusis in Tinnitus Individuals is Associated with Smaller Gray Matter Volumes in the Supplementary Motor Area Regardless of Hearing Levels

Punitkumar Makani [1,2,*], Marc Thioux [1,2,‡], Elouise A Koops [1,3], Sonja J Pyott [1,2,‡], and Pim van Dijk [1,2,‡]

[1] Department of Otorhinolaryngology–Head and Neck Surgery, University of Groningen, University Medical Center Groningen, Groningen, the Netherlands. p.makani@umcg.nl (P.M.); m.a.thioux@umcg.nl (M.T.); ekoops@mgh.harvard.edu (E.A.K.); s.pyott@umcg.nl (S.J.P.); p.van.dijk@umcg.nl (P.V.D.)

[2] Graduate School of Medical Sciences (Research School of Behavioral and Cognitive Neurosciences), University of Groningen, Groningen, the Netherlands. p.makani@umcg.nl (P.M.); m.a.thioux@umcg.nl (M.T.); s.pyott@umcg.nl (S.J.P.); p.van.dijk@umcg.nl (P.V.D.)

[3] Department of Radiology, Massachusetts General Hospital-Harvard Medical School, Boston, USA. ekoops@mgh.harvard.edu (E.A.K.)

* Correspondence: p.makani@umcg.nl (P.M.)

‡ These authors contributed equally to this work.

Supplementary Tables

Supplementary Table S1. Acquisition parameters for T1-weighted anatomical brain imaging.

	**Langers et al., 2012** [1]	**Boyen et al., 2013** [2]	**Amaral et al., 2015** [3]	**Koops et al., 2020** [4]
SENSE Coil Type	8-channel head coil	8-channel head coil	8-channel head coil	32-channel head coil
Repetition Time (ms)	9	9	9	10.4
Echo Time (ms)	3.6	3.5	3.6	5.7
Field of View (mm)	192 x 176	232 x 256	232 x 256	256 x 224
Flip Angle (°)	8	8	8	8
Number of Slices	144	170	170	160
Slice Thickness (mm)	1	1	1	1
Slice Gap (mm)	0	0	0	0
Scan Time (sec)	160	251	251	614

Supplementary Table S2. Overview of demographic, audiometric, questionnaires, morphometric data for the four participant groups.

Group	Normal Hearing and Tinnitus (NHT)		Hearing Loss and Tinnitus (HLT)		Statistic
	**Without Hyperacusis**	**With Hyperacusis**	**Without Hyperacusis**	**With Hyperacusis**	
**Demographic**					
n	13	22	41	25	-
Mean Age (years)	45.5 ± 11.2 [c,d]<br>(29 – 62)	44.1 ± 12.2 [c,d]<br>(19 – 59)	58.3 ± 10.5 [a,b]<br>(27 – 76)	59.5 ± 7.9 [a,b]<br>(41 – 73)	**$X^2(3) = 30.0$,<br>$p < 0.001$**
Sex (male ǀ female)	8 ǀ 5	10 ǀ 12	32 ǀ 9	15 ǀ 10	$X^2(3) = 7.0$,<br>$p = 0.071$
Handedness<br>(L ǀ R ǀ NP ǀ NA)	11 ǀ 2 ǀ 0 ǀ 0	21 ǀ 1 ǀ 0 ǀ 0	5 ǀ 29 ǀ 0 ǀ 7	0 ǀ 23 ǀ 2 ǀ 0	-
**Audiometric (for both ears 0.25 to 8 kHz)**					
Mean PTA (dB HL)	7.3 ± 5.4 [c,d]	9.4 ± 7.3 [c,d]	35.0 ± 8.6 [a,b]	38.2 ± 12.3 [a,b]	**$X^2(3) = 65.8$,<br>$p < 0.001$**
**Questionnaires**					
HQ	16.5 ± 3.6 [b,d]<br>(10 – 21)	27.1 ± 4.1 [a,c]<br>(22 – 35)	13.2 ± 5.4 [b,d]<br>(0 – 21)	26.2 ± 3.7 [a,c]<br>(22 – 33)	**$X^2(3) = 76.0$,<br>$p < 0.001$**
HQ-Attentional	5.3 ± 1.5 [b]	8.2 ± 1.8 [a,c]	3.7 ± 1.7 [b,d]	7.3 ± 1.4 [b]	**$X^2(3) = 56.0$,<br>$p < 0.001$**
HQ-Social	5.5 ± 2.1 [b,d]	10.7 ± 3.4 [a,c]	5.4 ± 3.4 [b,d]	10.5 ± 2.4 [a,c]	**$X^2(3) = 42.1$,<br>$p < 0.001$**
HQ-Emotional	5.7 ± 1.8 [b,d]	8.3 ± 2.1 [a,c]	4.1 ± 1.39 [b,d]	8.4 ± 2.1 [a,c]	**$X^2(3) = 48.1$,<br>$p < 0.001$**
HADS-Anxiety	6.8 ± 4.2<br>(0 – 16)	6.5 ± 3.4 [c]<br>(2 – 15)	3.7 ± 3.0 [b]<br>(0 – 11)	5.9 ± 4.6<br>(0 – 16)	**$X^2(3) = 11.0$,<br>$p = 0.011$**
HADS-Depression	5.0 ± 3.8<br>(0 – 14)	4.9 ± 3.7<br>(0 – 15)	3.2 ± 3.1 [d]<br>(0 – 10)	6.2 ± 4.7 [c]<br>(0 – 16)	**$X^2(3) = 8.4$,<br>$p = 0.038$**
THI	33.7 ± 20.4<br>(4 – 66)	44.4 ± 20.5 [c]<br>(10 – 88)	27.4 ± 19.3 [b,d]<br>(4 – 80)	41.8 ± 20.8 [c]<br>(6 – 82)	**$X^2(3) = 13.0$,<br>$p = 0.005$**
THI-Functional	18.9 ± 12.2	24.7 ± 10.4 [c]	15.6 ± 10.2 [b,d]	25.6 ± 11.8 [c]	**$X^2(3) = 15.0$,<br>$p = 0.002$**
THI-Emotional	7.5 ± 6.8	11.3 ± 8.5	6.7 ± 6.8	9.6 ± 6.9	$X^2(3) = 6.6$,<br>$p = 0.085$
THI-Catastrophic	7.2 ± 3.6	8.4 ± 5.1 [c]	5.2 ± 4.1 [b]	6.7 ± 3.9	**$X^2(3) = 8.0$,<br>$p = 0.045$**
**Morphometric**					
TIV (cm^3)	1429.6 ± 159.9	1419.1 ± 147.1	1494.9 ± 139.1	1451.0 ± 96.3	$X^2(3) = 4.1$,<br>$p = 0.248$

Mean ± Standard Deviation (Range). dB HL decibel hearing loss; HADS hospital anxiety depression scale; HLT participant group with tinnitus and bilateral sensorineural hearing loss (either with or without hyperacusis); HQ hyperacusis questionnaires; L left; NA not available; NHT participant group with tinnitus and clinically normal hearing (either with or without hyperacusis); NP no preference; PTA pure tone average (0.25 to 8 kHz); R right; THI tinnitus handicap inventory. [a]The group differed significantly ($p \leq 0.05$) from the NHT group without hyperacusis; [b]The group differed significantly ($p \leq 0.05$) from the NHT group with hyperacusis; [c]The group differed significantly ($p \leq 0.05$) from the HLT group without hyperacusis; [d]The group differed significantly ($p \leq 0.05$) from the HLT group with hyperacusis.

Supplementary Table S3. Results of Spearman's Rank correlation coefficients between the SMA gray matter volumes (HMAT and HBN atlas VOIs) and either the HADS anxiety and depression scores or the THI tinnitus burden scores across all participants. The statistical threshold for Spearman's Rank correlation coefficients was set at $p \leq 0.05$.The SMA VOIs (HMAT and HBN atlas VOIs) are shown in **Supplementary Figure S1**.

	**HADS Anxiety Score**	**HADS Depression Score**	**THI Tinnitus Burden Score**
**HMAT Atlas VOIs**			
SMA left	$r_s(100) = –0.05$, $p = 0.639$	$r_s(100) = –0.03$, $p = 0.722$	$r_s(99) = 0.09$, $p = 0.371$
SMA right	$r_s(100) = 0.04$, $p = 0.717$	$r_s(100) = 0.03$, $p = 0.805$	$r_s(99) = 0.04$, $p = 0.720$
**HBN Atlas VOIs**			
SMA_A6m left	$r_s(100) = –0.04$, $p = 0.686$	$r_s(100) = 0.01$, $p = 0.903$	$r_s(99) = 0.10$, $p = 0.303$
SMA_A6m right	$r_s(100) = –0.05$, $p = 0.612$	$r_s(100) = –0.01$, $p = 0.945$	$r_s(99) = 0.02$, $p = 0.875$
SMA_A4ll left	$r_s(100) = –0.04$, $p = 0.719$	$r_s(100) = –0.10$, $p = 0.284$	$r_s(99) = 0.05$, $p = 0.642$
SMA_A4ll right	$r_s(100) = 0.10$, $p = 0.338$	$r_s(100) = 0.03$, $p = 0.737$	$r_s(99) = 0.06$, $p = 0.564$

HADS Hospital Anxiety Depression Scale; HBN Human Brainnetome atlas; HMAT Human Motor Area Template atlas; SMA supplementary motor area; SMA_A6ma anterior subdivision of the supplementary motor area; SMA_A4ll posterior subdivision of the supplementary motor area; THI Tinnitus Handicap Inventory; VOIs volume-of-interests.

Supplementary Figures

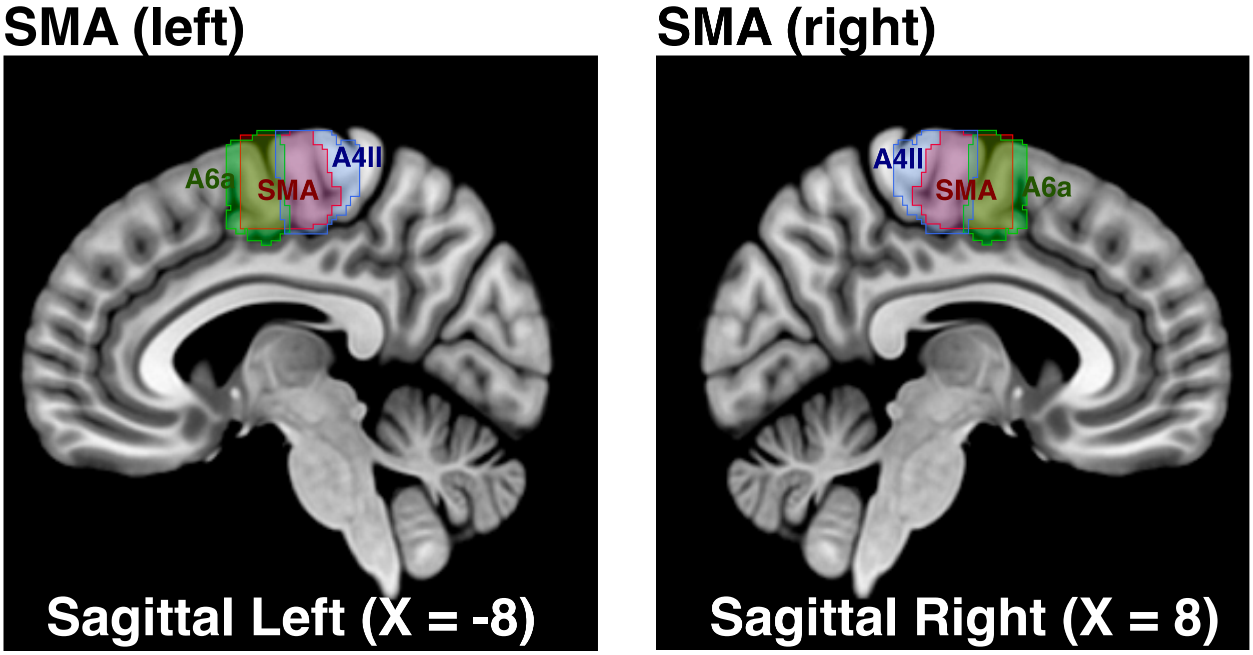

Supplementary Figure S1. We selected the volume-of-interests (VOIs) for the supplementary motor area (SMA) as defined in a previous study using both anatomical and functional MRI data [5]. These SMA VOIs (highlighted in red) are freely accessible as the Human Motor Area Template (HMAT) atlas from http://lrnlab.org/. We also selected VOIs for the anterior and posterior subdivisions of the SMA, labelled as SMA_A6m (highlighted in green) and SMA_A4ll (highlighted in blue) respectively, as defined by the structural and functional connectome architecture [6]. These SMA VOIs are freely accessible as the Human Brainnetome (HBN) atlas from https://atlas.brainnetome.org/bnatlas.html.

References

[1] D. R. M. Langers, E. de Kleine, and P. van Dijk, "Tinnitus does not require macroscopic tonotopic map reorganization," Front Syst Neurosci, vol. 6, p. 2, 2012, doi: 10.3389/fnsys.2012.00002.

[2] K. Boyen, D. R. M. Langers, E. de Kleine, and P. van Dijk, "Gray matter in the brain: Differences associated with tinnitus and hearing loss," Hearing Research, vol. 295, pp. 67–78, Jan. 2013, doi: 10.1016/j.heares.2012.02.010.

[3] A. A. Amaral and D. R. M. Langers, "Tinnitus-related abnormalities in visual and salience networks during a one-back task with distractors," Hearing Research, vol. 326, pp. 15–29, Aug. 2015, doi: 10.1016/j.heares.2015.03.006.

[4] E. A. Koops, E. de Kleine, and P. van Dijk, "Gray matter declines with age and hearing loss, but is partially maintained in tinnitus," Sci Rep, vol. 10, no. 1, Art. no. 1, Dec. 2020, doi: 10.1038/s41598-020-78571-0.

[5] M. A. Mayka, D. M. Corcos, S. E. Leurgans, and D. E. Vaillancourt, "Three-dimensional locations and boundaries of motor and premotor cortices as defined by functional brain imaging: a meta-analysis," Neuroimage, vol. 31, no. 4, pp. 1453–1474, Jul. 2006, doi: 10.1016/j.neuroimage.2006.02.004.

[6] L. Fan et al., "The Human Brainnetome Atlas: A New Brain Atlas Based on Connectional Architecture," Cereb Cortex, vol. 26, no. 8, pp. 3508–3526, Aug. 2016, doi: 10.1093/cercor/bhw157.
